# Improvement of Etching Anisotropy in Fused Silica by Double-Pulse Fabrication

**DOI:** 10.3390/mi11050483

**Published:** 2020-05-08

**Authors:** Valdemar Stankevič, Jonas Karosas, Gediminas Račiukaitis, Paulius Gečys

**Affiliations:** Center for Physical Sciences and Technology, Savanoriu Ave 231, LT-02300 Vilnius, Lithuania; jonaskarosas919@gmail.com (J.K.); g.raciukaitis@ftmc.lt (G.R.); p.gecys@ftmc.lt (P.G.)

**Keywords:** femtosecond, fused silica, double pulses, selective chemical etching

## Abstract

Femtosecond laser-induced selective etching (FLISE) is a promising technology for fabrication of a wide range of optical, mechanical and microfluidic devices. Various etching conditions, together with significant process optimisations, have already been demonstrated. However, the FLISE technology still faces severe limitations for a wide range of applications due to limited processing speed and polarization-dependent etching. In this article, we report our novel results on the double-pulse processing approach on the improvement of chemical etching anisotropy and >30% faster processing speed in fused silica. The effects of pulse delay and pulse duration were investigated for further understanding of the relations between nanograting formation and etching. The internal sub-surface modifications were recorded with double cross-polarised pulses of a femtosecond laser, and a new nanograting morphology (grid-like) was demonstrated by precisely adjusting the processing parameters in a narrow processing window. It was suggested that this grid-like morphology impacts the etching anisotropy, which could be improved by varying the delay between two orthogonally polarized laser pulses.

## 1. Introduction

Within the past two decades, femtosecond pulse processing of transparent materials has demonstrated outstanding results [1,2]. Many various processing technologies were developed for marking, dicing, cutting and internal modifications of transparent materials. Femtosecond laser-induced selective etching (FLISE) was demonstrated for the first time in 2001 by Marcinkevičius et al. when a track recorded by a femtosecond laser inside fused silica was selectively etched along the modified region [3]. That was the start of subtractive manufacturing in transparent materials, mainly in bulk fused silica by forming microchannels and complex 3D structures. It was found, within two years, that the modifications are composed of self-organised nanogratings oriented perpendicular to the laser polarisation [4]. The detailed investigations showed that selective etching is related to the nanograting orientation [5] and molecular oxygen in nanopores within the nanogratings [6]. The latter discoveries prompted many FLISE optimisation works to be conducted [7,8,9,10], and FLISE has become a very promising technology for a wide range of complex applications, such as internal 3D structures for mechanical, optical and microfluidic devices [11,12,13]. The most used and investigated material for laser-induced chemical etching is fused silica. 

New opportunities came from double-pulse fabrication approaches recently introduced for the processing of various materials. Double pulses with different wavelengths [14] or double-pulse laser technology for material ablation [15] gained the increased attention for the processing of transparent materials [16]. Pengjun et al. demonstrated that the temporal shaping of the femtosecond pulses could provide higher etching selectivity, and, at some defined pulse energies, the etching rate could be a few times higher compared to conventional single pulses [17] due to the better efficiency of photon absorption. The promising results were also achieved by combining double pulses with a linear and circular polarisation where the improvement in the etching rate for the formation of high aspect ratio channels was demonstrated [18]. In most publications it is assumed that the first pulse is responsible for the nanograting orientation [14,19]. However, the real situation could be more complicated. The nanograting formation in fused silica is usually related to the enhanced birefringence. Atoosa et al. demonstrated that orthogonally-polarised double-pulses could reduce the birefringence because the nanograting orientation is determined by the writing pulse with a higher intensity [20]. More recent work also confirmed that the second pulse could rewrite the nanograting orientation when the double pulses with non-equal energies are applied [21]. However, there are still limitations of the FLISE technology due to etching anisotropy (etching rate dependence on laser writing direction at a constant linear polarisation) and low processing (laser writing and etching) speed. The efforts to overcome this drawback was already made for single-pulse processing by varying the polarization direction during fabrication [22], or by changing laser pulse duration to the picosecond range [23]; however, the problem was not solved completely.

In this work, we investigate the double-pulse processing approach with crossed polarisations and variable inter-pulse delay. Initially, peculiarities of nanograting morphologies recorded with double pulses were investigated. Hereafter, we show the influence of the pulse duration and inter-pulse delay to the selective etching of the microchannels. In most applications, the laser writing trajectory has a curved shape. The nanogratings are statically orientated along the curved patch. Therefore, at different trajectory position, the nanograting orientation is shifted relative to the trajectory vector, and, as a consequence, the orientation-dependent etching takes place. To overcome this drawback, the double pulses with crossed polarisations were used, and the etching anisotropy and etching rate improvement were demonstrated. Particular experiments were arranged to fabricate the vertical bow-like structures—two-dimensional structures formed by raster scanning of a bow-like horizontal shape starting beneath the sample bottom surface and moving laterally through the laser focus up to the top sample surface. To our best knowledge, we demonstrate the new grid-like nanograting morphology for the first time and the possible explanation provided. That is an entirely new phenomenon that was not described earlier.

## 2. Materials and Methods

**Figure 1:**
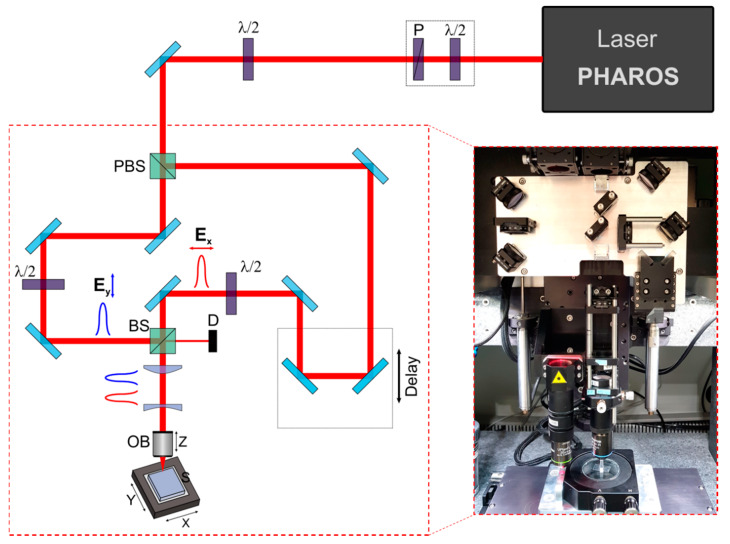
The double-pulse experimental setup. P—Brewster angle polarizer, PBS—polarising beam splitter, BS—beam splitter, D—dump, OB—microscope objective.

The setup for double-pulse experiments is schematically illustrated in Figure 1. The micromachining workstation with an integrated femtosecond laser (Pharos-6W, Light Conversion, Vilnius, Lithuania) was used. The ultrashort laser operated at a 1030 nm wavelength, and the 500 kHz pulse repetition rate was mainly used during the experiments. The laser was equipped with a tunable compressor which allowed the pulse duration to be tuned in the range of ~0.26 to ~10 ps with a positive or negative chirp. The laser power was controlled using an external attenuator which was calibrated to vary the mean laser power linearly. The laser beam was split into two pulses with a polarising beam splitter (PBS). The energy ratio between two pulses was controlled by rotating a λ/2 phase plate. During the tests, the energy ratio was set to 1:1. The Mach–Zehnder interferometer setup, composed from a variable arm (VA) and reference arm (RA), was used to combine two laser beams with a controllable temporal delay between pulses. The polarisation in the reference arm was set to ***E_y_*** and that in the variable arm to ***E_x_***. Afterwards, the beam diameter was reduced to ~2 mm using a two-lens telescope system and focused to the sample using the 100× microscope objective (M Plan NIR, Mitutoyo, Kanagawa, Japan) with a numerical aperture (NA) of 0.5. The microscope objective was translated with a linear translation stage (ANT130-L, Aerotech, Pittsburgh, PA, USA) along the Z direction. The 20× camera vision objective (Olympus Plan Achromat, Tokyo, Japan) was mounted on the same stage. A sample was mounted on a two-axis gimbal mount (GM200, Thorlabs, Mölndal, Sweden) and translated by a high-resolution (>500 nm) XY linear stage (ANT130-XY, Aerotech, Pittsburgh, PA, USA) with a scan speed up to 5 mm/s. The translation stages were controlled via the controller (A3200, Aerotech, Pittsburgh, PA, USA).

**Figure 2:**
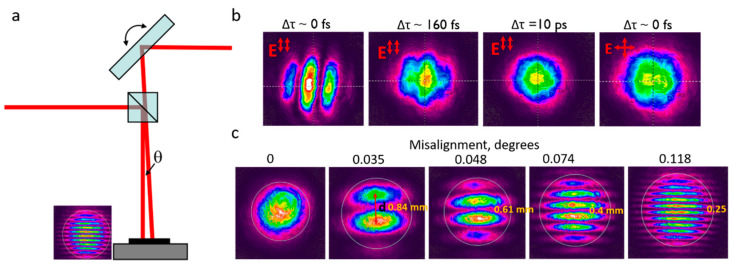
Spatial and temporal beam alignment: (**a**) two-beam angle adjustment principle; (**b**) intensity profiles of two beams with different temporal delay and polarisation orientation; (**c**) interference patterns of two beams with various misalignment angles at the 0 fs delay.

The delay range from –20 ps to 20 ps was possible to achieve, between the two pulses, a temporal delay resolution of ~7 fs. The delay was estimated relative to the reference arm; consequently, the negative delay was achieved when the variable arm was shorter with respect to the reference arm (the reference-arm pulse was the second). During the tests, the delay was varied from −10 ps to +10 ps. In further text, to avoid confusion, the following indication will be used: ***E*_‖_**: polarization parallel to the scanning direction, and ***E_⊥_***: polarization perpendicular to the scanning direction. The pulse order is indicated as the first or second one. To obtain the 0 fs delay, the double-pulse setup was calibrated temporally (Figure 2). Initially, the temporal delay was calibrated by changing the length of the delay line with a slight angular misalignment between the beams and registering the intensity profile with a CCD camera (Spiricon SP620U, Ophir, Jerusalem, Israel). When the delay in the variable arm approached the ~0 fs delay (i.e., two laser pulses overlap entirely in time), the clear interference intensity pattern was registered. When the laser pulses overlapped only partly (~160 fs), the interference was still observed. However, the contrast was very poor. For relatively long delays (>300 fs), the laser beams did not interfere. The interference condition also was not satisfied for cross-polarised beams, and the non-interfering double-pulse beam profile was observed even at the 0 fs pulse delay.

The spatial beam position adjustment was performed by recording the interference pattern at the 0 fs delay and measuring the interference period. According to the relation of the interference pattern and the beam angle, *d* = *λ*/2*sinθ*, it was possible to calculate the angle between two beams. The interference period increased when the angle decreased. Therefore, two beams appeared almost parallel after a few iterations of mirror angle adjustment.

**Figure 3:**
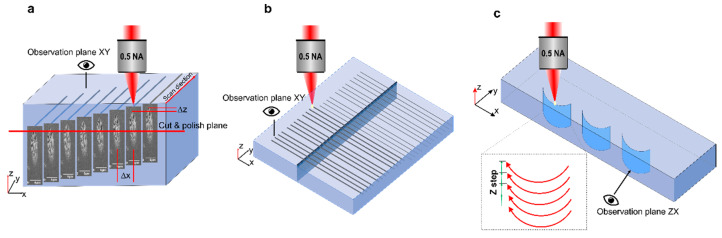
Schematic illustration of experiments: (**a**) recording and polishing of the lines written with double pulses for nanogratings observation by scanning electron microscope (SEM); (**b**) the line writing procedures for the investigation of the double-pulse influence on the chemical etching rate; (**c**) recording of the vertical bow-like structures for the investigation of directional etching dependence.

Commercially available fused silica (JGS1, 20 mm × 3 mm × 2 mm, Eksma Optics, Vilnius) samples were used for the experiments. The modifications were recorded by focused laser beam ~20 µm below the sample surface at the regimes when nanogratings were formed (Type II modification) [24]. A few parallel lines shifted by a z-step (1 µm) were recorded to ensure that the right vertical cross-section of the nanogratings would be investigated after the cutting and polishing procedure (Figure 3a). The exposed nanogratings were etched for 1 min in 5% hydrofluoric acid (HF) acid and coated with a ~10 nm gold layer. The prepared samples were investigated by scanning electron microscope (SEM) (JEOL, JSM6490LV, Tokyo, Japan).

To investigate the double-pulse delay effects to the chemical etching rate of the fused silica samples, a group of lines was written by varying the pulse energy and scan speed at a constant repetition rate. The separate lines were recorded at different focusing depth (50–500 µm below the sample surface) with the vertical raster-scanned surface ending at the sample top surface in the middle of the recorded track to get the acid access to the modified area (Figure 3b). Three lines in each group were recorded under the same conditions and the measured data were averaged. The directional etching dependence on the double-pulse delay was investigated recording the bow-like vertical structures (Figure 3c) by a raster scanning the single bow-like (curved) trajectory laterally moving through the laser focus with a 7 µm vertical z step, starting beneath the bottom sample surface and ending on the sample top surface.

After the irradiation with the focused ultrashort laser pulses, the fused silica samples were immersed into a wet-etching bath of HF with a concentration of 10% and 30 °C temperature for 30 min. After the chemical etching, the samples with the vertical bow-like structures were polished in the XZ plane with a 0.3 µm grade colloid silica to recover a smooth, transparent surface for the high-contrast microscope measurements.

## 3. Results and Discussion

### 3.1. Etching Rate of Modifications Recorded with Double Femtosecond Pulses

Multiple lines were inscribed in the bulk of fused silica, as shown in Figure 3a to investigate the effects of the inter-pulse delay, pulse energy, and focusing depth to the etching rate of the double-pulse modified material. The group of lines were recorded with different pulse density ranging from 100 to 2000 ppµ (pulses/µm), keeping the laser pulse energy constant. Various laser pulse energies were applied from 100 nJ to 600 nJ for each group of lines. It should be noted, that the indicated pulse energy was measured when two pulses were combined into one optical path, so the pulse energy for separate beams was twice lower. That was done to allow a direct comparison of the results with the single-pulse processing. In the single-pulse processing experiments, one of the beams was blocked.

**Figure 4:**
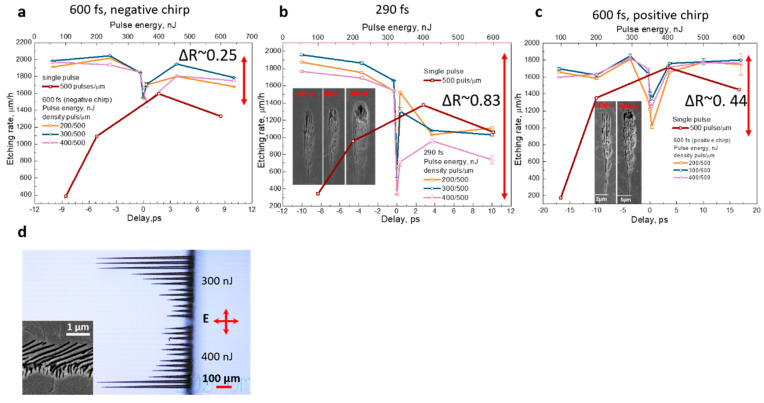
The etching rate dependence on the delay between two pulses for modifications recorded in fused silica with the 200–400 nJ pulse energy and orthogonal polarisations: (**a**) 600 fs pulse duration with a negative chirp; (**b**) 290 fs pulse duration; (**c**) 600 fs pulse duration with a positive chirp; (**d**) The microscope picture of etched channels for the 0 fs pulse delay at 290 fs pulse duration. Red curve: the etching rate dependence on the pulse energy for a single pulse. Samples were etched 30 min in 10% diluted hydrofluoric acid (HF) acid. The inset shows the SEM pictures from the side (**b**,**c**) and top (**d**).

In our previous works, the maximum etching rate of the fused silica was ~1300 µm/h with the leading etching selectivity of ~120:1, when processing with the 1030 nm wavelength was applied [25,26]. The results were achieved with ~400–600 nJ pulse energy and 1000 ppµ density. The results of the double-pulse processing in fused silica are demonstrated in Figure 4. The visual observation showed that even for double-pulse processing, the pulse duration had a strong influence on the etching rate. At 0 fs pulse delay and various pulse durations, we observed the etching rate variation when the pulse density was changed. A lower etching rate variation was observed for longer pulse durations independently on the chirp direction.

The more surprising result was observed when the pulse delay was varied from −10 ps to +10 ps. As demonstrated in Figure 4a–c, the etching rate significantly reduced when the delay between two pulses approached 0 fs. For the positive and negative delays, the etching rate grew almost symmetrically when the pulse duration was 600 fs. For the shortest pulse duration (~290 fs with compensated chirp) and negative delays, the etching rate increase was higher compared to the positive delays. In the case of negative delay, the first beam coming from the variable arm was with the parallel polarisation (***E*_‖ first_**), and only then the second beam from the reference arm with the perpendicular polarization (***E*_*⊥* second_**) was arriving. The etching rate difference could be related to the different modification thresholds for the nanograting formation, depending on the beam polarisation [6]. Therefore, the significant influence of the second pulse (***E*_*⊥* second_**) to the nanogratings orientation in the negative delay range was observed. Saturation of the etching rate was observed when the pulse delay approached ~10 ps independently on the delay direction. More objective etching rate comparisons could be made by introducing the etching rate contrast parameter Δ*R*, which is described as the ratio of the difference between the maximum and minimum etching rate and maximum etching rate as follows Δ*R* = (*R_max_* − *R_min_*)/*R_max_*. According to this description, the etching contrast for the 600 fs pulse duration was consequently Δ*R*_−600fs_ = 0.25 and Δ*R*_+600fs_ = 0.44. The maximum etching rate contrast was achieved for the shortest pulse duration Δ*R*_290fs_ = 0.83. The etching rate contrast showed how much the pulse delay influenced the etching rate: the lower etching rate contrast value, the weaker the pulse delay influence on the etching rate was observed. For longer pulse durations, the broader pulse delay range with a lower etching rate was found compared to the shorter pulse duration. Longer pulses had a wider temporal overlap range that confirms the observations of the highest etching rate drop only in the area where the temporal pulses overlap. It could be noted that the etching rate contrast drop was usually observed for higher pulse energies. By comparing the etching results of the single-pulse and double-pulse experiments, we can distinguish a significant difference in the etching rate. While for the single-pulse processing at the same pulse energy, the 1300–1400 µm/h etching rate was the maximum value that could be achieved, the double-pulse processing enhanced the etching rate up to 2000–2100 µm/h. That yielded >30% of the etching rate increase. This achievement is even more attractive as this rate enhancement can be achieved with a lower pulse density of 100–500 ppµ (pulse per micrometre), which allowed us to speed up the processing more than two times. Murata et al. demonstrated that for the double-pulse processing, the diameter of emerging nanopores is twice time larger comparing to the single-pulse processing [27]. We believe that such behaviour had substantial input to the etching rate increase in our case due to the higher area of nanopores.

**Figure 5:**
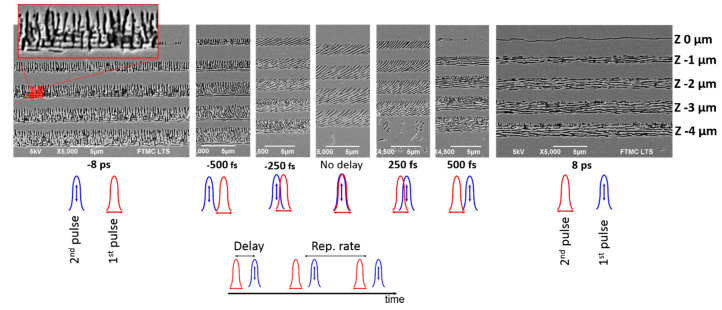
SEM pictures of the nanograting morphology evolution depending on the delay between two pulses. The inset shows the enlarged area of the grid-like nanograting structure. The nanostructures recorded with a total 400 nJ pulse energy (200 nJ + 200 nJ).

To understand the etching rate enhancement, the in-depth investigations of the Type II modifications induced using double pulses at various delay times were performed. Figure 5 shows the nanograting morphology dependence on the delay between two pulses.

For the 0 fs delay between two pulses, the nanogratings consisted of parallel lines rotated by ~45° relative to the scanning direction. When temporal pulse overlap was in the range of laser pulse duration (±250 fs, for ~290 fs pulse duration), the 45° nanograting orientation was still observed. The nanogratings were slightly shifted from the straight line and appeared bow-shaped indicating the nonperfect spatial beam overlap. When the positive delay exceeded the pulse duration, the evident influence of the first pulse (***E*_*⊥* first_**) on the nanograting formation was observed, that confirmed the results described by Rohloff et al. [28]. They found that, at low fluences, the orientation of the laser-induced periodic surface structures (LIPSS) structures on the fused silica surface changed their direction by 90° when delay direction changed, and it was determined by the first pulse polarization.

In our case, when the delay between laser pulses approached to −10 ps (***E*_‖ first_**), the grid-like structures were observed, which was an unexpected result considering the previous investigations [19,29]. This result was replicated a few times and was repeatable. To our best knowledge, the grid-like structures in the bulk fused silica were demonstrated for the first time, and there are no valuable phenomena to explain. We can speculate that the mentioned structures appear as a consequence of the different modification thresholds for two polarisations. According to previous investigations, the Type II modification threshold for perpendicular polarisation is ~2 times lower compared to the parallel polarisation [6]. The electron plasma generated by the first pulse absorbs more efficiently the second pulse due to the reduced ionisation threshold [30]. As a consequence, the already created point defects, such as colour centres (E’) and nonbridging oxygen hole centres (NBOHCs) [31], interact with the electrical field of the second pulse. Such interaction could enhance the electron plasma more effectively due to the lower modification threshold for perpendicular polarisation and a memory effect [30]. However, as it is known, at least a few tens of pulses are required to rewrite the nanogratings [32]. The polarisation changed after each pulse in the double-pulse regime, and that was not sufficient to overwrite the nanograting orientation. When the delay approached the −10 ps, the orientation of the inhomogeneities was created and defined by the electrical field orientation of the first pulse (***E*_‖__first_**) due to formed STE (self-trapped excitons) and not relaxed states still at a high temperature which involves the viscous flow of the silica. Hence, the induced periodic electron plasma pattern by the second pulse (***E*_*⊥* second_**) with the perpendicular polarisation records the periodical nanopattern on top of the already oriented nanogratings partly covering them due to the lower modification threshold.

From another point of view, according to the numerical solution of Maxwell’s equation in the vicinity of subsurface planar cracks oriented normal to the surface of fused silica [33] (created nanogratings in our case), the higher field enhancement is predicted (approx. by a factor of 2) inside narrow cracks (nanoplates) which are oriented perpendicular to the laser polarisation. In the case of negative delay (***E*_‖ first_**), the more significant field enhancement is predicted for the first pulse (***E*_‖ first_**) with the parallel polarisation (it first creates nanogratings perpendicular to the polarisation). However, due to a higher modification threshold for the same polarisation, the second pulse (***E*_*⊥* second_**) should generate a higher amount of free electrons and create the grid-like nanostructures. In the case of positive delays (***E*_*⊥* first_**), the influence of the first pulse strongly prevails due to higher light enhancement factor for the first pulse polarization (it first creates nanogratings perpendicular to the polarization) and lower modification threshold. Therefore, only a small part of the second pulse (***E*_‖ second_**) induced nanogratings is noticeable. However, for longer delays, the second pulse (***E*_‖ second_**) could have a stronger influence. As follows from Figure 4, the reduced etching rate for positive delays was observed.

The mechanism of the tilted nanogratings for the 0 fs delay can be simply explained in the following way: for spatially and temporarily adjusted beam the nanogratings are ~45° tilted relative to the X or Y polarisations, this is caused by the superposition of two pulses with orthogonal polarisations with no phase delay between two pulses, and the resultant vector is rotated by 45° [34,35]. The smallest step size of the delay line micrometre stage was 1 µm that changed the length of the delay line for double-length and corresponded to the ~7 fs delay. The resolution is not sufficient to precisely control the phase delay between two pulses, however for the exceptional case of 0 fs delay, the phase between two pulses could be set accurately due to the registration of the interference pattern of the slightly misaligned pulses. In this case, even the phase delay is set not accurately, and the elliptical polarization dominates, the morphology of the nanogratings still was normal to its major axis [36].

**Figure 6:**
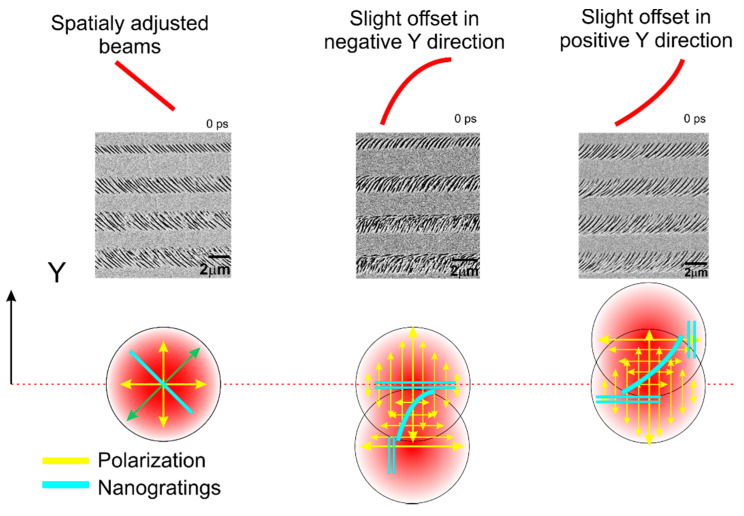
The pictorial explanation of the nanograting shape only for temporally adjusted pulses (0 fs delay) when a slight spatial beam misalignment is induced in the Y direction.

For the small beam misalignment in the +Y or −Y directions, the nanogratings were of the bow shape (Figure 6) due to partial interaction of the beams and domination of the opposite polarisations in the modification of top and bottom areas. For the intermediate case, the nanogratings were the result of both beams. For longer delays, the pulses did not interact coherently. Peculiarities in the nanograting morphology were defined by the interaction of the second pulse with the material excited by the first pulse.

### 3.2. Directional Etching of Vertical Bow-Like Structures

In previous sections, we discussed the etching of the single microchannels recorded with the polarisation normal to the scan direction or utilising double-pulses with orthogonal polarisations. To investigate the double-pulse effect to the writing direction, bow-like vertical structures were inscribed in fused silica (Figure 3c). This way, the beam polarisation angle was constantly shifting relative to the writing direction of the bow-like curvature. Therefore, the different etching rate was obtained for the single-beam case depending on the location on the curve [22]. We were expecting that double pulses with orthogonal polarisations could suppress this effect due to grid-like structure formation and enhanced nanopores formation [27]. It should be considered, that in this recording configuration, additional influence of the line stacking along the beam propagation direction had an impact on the etching rate.

**Figure 7:**
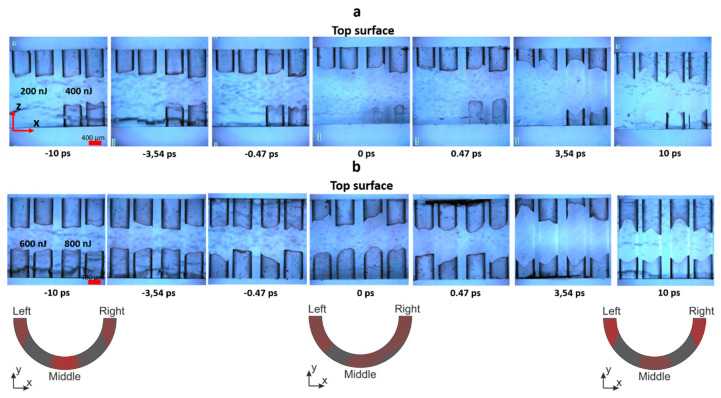
The microscope measurement of the etching dependence on the double-pulse delay for the bow-like vertical structures from the sample side (ZX plane): (**a**) the structures recorded with 200 nJ and 400 nJ pulse energy and (**b**) the structures inscribed with 600 nJ and 800 nJ pulse energy. Two channels with 500 and 1000 ppµ density were recorded at the constant pulse energy. Etching performed for 30 min. in 10% HF. The inset shows the bow-like trajectory view on the XY plane with a predicted nanogratings orientations.

**Figure 8:**
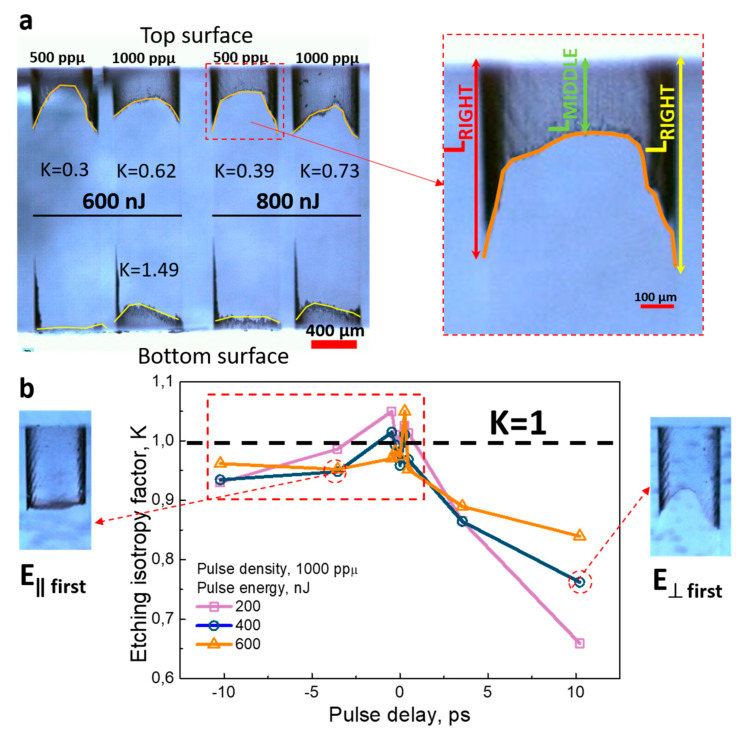
Etching of bow-like structures fabricated with the single-pulse regime at 600 nJ and 800 nJ pulse energy (**a**) and dependence of the etching isotropy factor *K* on the pulse delay for etching from the top sample surface of the vertical surface. (**b**). The insets show the microscope pictures with etched vertical surfaces.

The vertical bow-like structures were recorded with the different double-pulse delay from −10 ps to +10 ps in fused silica. The separation between single vertical lines was set to 7 µm and was constant during all experiments. For one set of the double-pulse delay, an array of vertical surfaces with two different pulse densities of 500 and 1000 ppµ and four different pulse energies 200, 400, 600 and 800 nJ were recorded. The structures were etched 30 min in 10% HF acid. The etching behaviours of the vertical structures are demonstrated in Figure 7. Due to disturbance in the laser beam profile by the spherical aberration, a weak sample etching from the bottom surface was observed for the lower pulse energies (200–400 nJ). For higher pulse energies, the etching rate from both bottom and top surfaces was comparable at a delay in the range from −10 ps to 0 ps. However, the bottom structure etching rate was lower at the delay from 0 ps to 10 ps. The etching dependence on the direction was minimised for negative delays (***E*_‖ first_**) (for etching from the top and bottom surfaces). In the case of positive delays (***E*_*⊥* first_**), the directional etching effect was significant, demonstrating the lower etching in the middle part of the vertical structure (Figure 8b, left inset).

For a more objective analysis, we introduce the etching isotropy factor *K*, which demonstrates the etching difference of the bow-like vertical structures in the middle part and corner parts (Figure 8a) *K* = 2*L*_M_/(*L*_R_ + *L*_L_), where *L*_M_, *L*_R_ and *L*_L_ are consequently the etched depth at the middle, right and left structure part. When *K* is approaching 1, uniform etching is obtained. For comparison, we recorded the bow-like vertical structures with a single pulse (Figure 8a). Usually, for this regime, the etching isotropy factor was below 0.7 and going down when the pulse energy or pulse density was decreased. For the 600 nJ pulse energy, the etching isotropy factors for single and double-pulse recording were 0.62 and 0.84, respectively. The 26% gain in the etching isotropy was obtained. The etching isotropy factor increased by raising the pulse energy for the positive pulse delays (***E*_*⊥* first_**) (Figure 8b). However, *K* > 1 value was observed in the delay range from −0.3 ps to 0.3 ps, i.e., within the temporal pulse overlap range. The etching rate measurement also demonstrated that the maximal etching rate of ~ 1500 µm/h was achieved usually for negative delays (***E*_‖ first_**). The difference in etching rate was marginal and close to the maximal value for the positive delays (***E*_*⊥* first_**) in some cases. In comparison for the single-pulse regime, the maximal achieved etching rate was ~1000 µm/h, which is ~33% slower and agrees well with the single channels etching results. Therefore, the nanograting orientations in the double-pulse regime played a significant role and enabled easier etchant penetration to the modified area. The etching behaviour for different delays could be confirmed by the nanograting investigation in Figure 5, where the grid-like nanogratings were demonstrated. On the left or right side of the bow-like structure, the speed vector made up the different angle with the polarisation. When on the middle part the first pulse polarisation is parallel (***E*_‖ first_**), on the left or right side it tends to be perpendicular, that made the nanogratings to be along the scan direction. That behaviour suppressed the directional etching dependence. Contrary, in the positive delays (***E*_*⊥* first_**), the first pulse influence should be leading in the middle part of the surface, where the nanogratings along the scan vector should be formed, consequently at right and left side the grid-like nanogratings were pronounced. According to this description, the faster etching rate was predicted in the middle structure part, as we can observe for the pulse delays from 0 ps to +0.47 ps. However, for the longer positive delays, the opposite behaviour was found. We could speculate, that the second pulse (***E*_‖ second_**) also had a substantial impact on the nanograting formation, where the middle structure part was less etched. Especially the second pulse influence to the nanogratings direction was observed for the longer delays (>10 ps) and higher second pulse energy [21].

## 4. Conclusions

The etching peculiarities of modifications in the fused silica recorded with the variable delay double-pulses were observed and discussed. The double-pulse fabrication enhanced the etching rate by >30% compared to the single-pulse fabrication. The detailed analysis of the nanostructures in the fused silica revealed the appearance of the grid-like nanostructures when the first pulse polarization was along scan direction (***E*_‖ first_**, negative delay). The nanogratings morphology explains the etching rate dependence on the pulse delay. The etching rate raised for negative and positive delays and was suppressed at the 0 fs delay, where the 45° oriented nanostructures are formed. The application of double pulses significantly improved the etching isotropy of the vertical bow-like structures. It was measured qualitatively by introducing the etching isotropy factor that showed the value near 1 for negative delays. We demonstrated that the double-pulse processing technique is a simple fabrication method that improves the etching isotropy without a need for phase plate rotation during processing. The etching rate improvement is even more attractive as this rate enhancement can be achieved with lower pulse density of 100–500 ppµ, which allows to speed up the processing more than two times.
